# Antral follicle responsiveness assessed by follicular output RaTe(FORT) correlates with follicles diameter

**DOI:** 10.1186/s13048-019-0522-4

**Published:** 2019-05-25

**Authors:** Camila Bessow, Rafaela Donato, Tatiane de Souza, Rita Chapon, Vanessa Genro, João Sabino Cunha-Filho

**Affiliations:** 1 Universidade Federal do Rio Grande do Sul, Rua Ramiro Barcelos, 2350, Porto Alegre, Brazil; 2 Hospital de Clínicas de Porto Alegre, Rua Ramiro Barcelos, 2350, Porto Alegre, Brazil; 3 Centro de Reprodução Humana Insemine, Porto Alegre, Brazil

**Keywords:** Follicular output rate, FORT, Controlled ovarian hyperstimulation, Antral follicle size, Ovarian responsiveness

## Abstract

**Background:**

The antral follicle count is a marker of ovarian reserve. Follicular Output RaTe (FORT) evaluates the proportion of follicles responsive to exogenous follicle stimulating hormone (FSH) during controlled ovarian stimulation. Our objective was to evaluate whether the diameter (AFC6: ≤ 6 mm or AFC > 6: > 6 mm) of the follicular cohort could be a predictor for ovarian responsiveness, assessed by FORT, in a prospective cohort with 92 women with IVF indication, regular cycles and no abnormality in both ovaries.

**Results:**

The mean age (±SD) of the women was 36.03 years (± 3.87 years), the median FORT was 43.30%. We found correlation between the FORT and AFC6 (r = − 0.237, P 0.023) but not between the FORT and AFC > 6 (r = − 0.055, P 0.602).

**Conclusions:**

The inverse correlation between FORT and AFC6 suggests that those follicles were less responsive to the exogenous FSH.

## Background

Follicular recruitment and development in response to controlled ovarian hyperstimulation (COH) with gonadotropins are essential in the treatment of infertility with assisted reproductive techniques [1]. The prediction of this ovarian response, which is evaluated through the ovarian reserve, is fundamental for both prognosis and treatment individualization.

Treatment individualization entails selecting the best GnRH analogue protocol and the best initial gonadotropin dose to achieve the ideal ovarian response because both low and hyper-responses are known to potentially lead to cycle cancellation and increased costs [2]. While a low ovarian response may decrease the chance of pregnancy, an exaggerated response presents the risk for the development of ovarian hyperstimulation syndrome [3–5].

Ovarian reserve has complex mechanisms that can be influenced by age, genetics and environmental factors [6]. Several markers of ovarian reserve have been studied in the last few decades, but an ideal marker still has not been established. Response prediction through ovarian reserve testing is superior to that through chronological age alone. The antral follicle count (AFC) and anti-Müllerian hormone (AMH) are currently considered to have the best sensitivity and specificity to predict the ovarian response, despite them having 10–20% false-positive rates [7]. Since ovarian responsiveness seems to be influenced by many factors, nomograms, based mostly on the age and AMH, AFC and day 3 FSH levels of women, were developed in the past few years to determine the individualized FSH starting dose in IVF cycles to reduce costs and improve the chances of pregnancy [8, 9].

Ovarian responsiveness is one of the most commonly studied parameters in clinical research concerning IVF treatment [10]. Traditionally, the number of oocytes retrieved is the main outcome measure of ovarian responsiveness to gonadotropin stimulation [5]. However, the number of pre-ovulatory follicles obtained at the end of COH is not a reliable reflection of the antral follicle sensitivity to FSH, and it is strongly correlated with the number of antral follicles before ovarian stimulation [11]. Although, maybe not only the number of antral follicles, but also their size, is important. The Follicular Output RaTe (FORT) evaluates the proportion of follicles that were responsive to FSH and is calculated by dividing the number of preovulatory follicles (16–20 mm) × 100 by the antral follicle count (3–8 mm), and it is known to be correlated to the outcomes of IVF, including pregnancy rates [12].

The distinction of various size categories may be relevant since several studies show that the endocrine function of a follicle is related to its diameter. [13, 14]. A previous study reported that pregnant patients had the highest numbers of antral follicles between 5 and 10 mm. [15]. Another study demonstrated that the number of follicles of 2–6 mm declines with age but that the number of follicles of 7–10 mm remains stable, suggesting that follicles smaller than 6 mm may represent the functional capacity of the ovary [14]. Mechanisms of the sensitivity of antral follicles to FSH remain to be completely elucidated. However, an adequate responsiveness to FSH is a characteristic of healthy and differentiated granulosa cells [16, 17].

The main objective of this study was to evaluate whether the pool of follicles up to 6 mm or larger than 6 mm correlates better with the ovarian response to the controlled stimulus, evaluated through the FORT.

The secondary objectives of this study were to evaluate the correlation among the FORT and other factors that can influence ovarian responsiveness, such as age, body mass index (BMI), and the levels of AMH and FSH.

## Materials and methods

### Subjects

We prospectively studied 92 patients, ranging from 26 to 45 years of age, who underwent COH for IVF + embryo transfer (ET) from January 2015 to August 2017. All of the patients met the following inclusion criteria: 1) both ovaries were present, morphological abnormalities (such as cysts, endometriomas, etc.) were absent, and transvaginal ultrasound scans having adequate visualization; 2) regular menstrual cycles lasting between 25 and 35 days. The exclusion criteria were: 1) current or past diseases affecting the ovaries or those affecting the secretion, clearance, or excretion of gonadotropin or sex steroids; 2) clinical and/or biological signs of hyperandrogenism; 3) diagnosis of polycystic ovarian syndrome.

Local institutional review board approvals for the use of clinical data for research studies were obtained and written informed consent was obtained from all patients.

### COH and IVF-ET protocol

Patients were submitted to COH with a flexible GnRH antagonist protocol [18]. In brief, between day 1 and day 3 of the menstrual cycle, the gonadotropin, either Elonva® 100 mcg or 150 mcg (Alfacorifolitropina, Merck & Co), was administered. The choice of the gonadotropin dose was decided based on patient’s weight: 100 mcg was selected for patients weighing less than 60 kg, and 150 mcg was selected for patients weighing 60 kg or more. According to the follicular response and after the seventh day of the COH, Menopur® (human menotropin gonadotropin, hMG, Ferring) was used if necessary (75–300 IU). Orgalutran® 0,25 mg (GnRH antagonist, Schering Plough) was started when a follicle reached 12 mm. Choriomon® 5000 IU (Human chorionic gonadotropin, hCG, Meizler) was administered as soon as ≥2 preovulatory follicles (16–22 mm in diameter) were observed. Oocytes were retrieved by transvaginal ultrasound- guided aspiration 36 h after hCG administration. The luteal phase was supported with 600 mg/day micronized progesterone administered continuously by the vaginal route, starting on the evening of the embryo transfer. The embryo(s) transfer was performed on day 2, 3 or 5, depending on embryo development. Clinical pregnancy was defined as the presence of an intrauterine embryo with cardiac activity at around 6 weeks after treatment.

### FORT calculation

The calculation of the FORT is also explained elsewhere [12]. The FORT was calculated by using the ratio between the number of preovulatory follicles (PFC) on hCG day × 100 and the number of AFC at baseline. The choice of considering only the 16–22 mm follicles for the calculation of the FORT was determined in a previous investigation by our group [13] and represented a methodological attempt for discriminating the follicles that were the most FSH-responsive among the cohort of small antral follicles.

### Hormonal measurements

Blood was collected by venipuncture on day 1–3 of a preceding cycle (1–3 months prior to the IVF procedure). Serum AMH levels were determined using an ultrasensitive enzyme-linked immunosorbent assay (Beckman Coulter, Inc., Brea, CA, USA), with sensitivity < 0.02–18.4 ng/mL and the maximum intra- and inter- assay imprecision (CV) were 12 and 14%, respectively.

Serum FSH levels were determined using a chemiluminescence immunoassay (Siemens Om-MA Immulite 2000, Munich, Germany). The range of detection of the immunoassay were 0.3–200 IU/L, and intra-and inter-assay CVs were 3 and 5%, respectively.

### Ultrasound measures

The antral follicular size and count assessments were performed with a 5–6 MHz transvaginal probe Famio 5 (Toshiba, Japan) prior to gonadotropin administration on menstrual days 1–3. The antral follicle count was defined as the number of follicles with a diameter of 3–8 mm. The follicular size was given as the mean of the two largest diameters in the same plane and perpendicular to each other. In this paper, our baseline AFC count was divided into follicles with a diameter of 6 mm or less and those with a diameter greater than 6 mm. The follicles with a diameter of 6 mm or less are represented by AFC6, and the follicles with a diameter larger than 6 mm and up to 8 mm were represented by AFC > 6.

### Statistics

The measure of central tendency used for parametric data was the mean, and the measure of variability used was the standard deviation (SD). The median and the minimum and maximum values were used when normality could not be ascertained. The Gaussian distribution was assessed by the Shapiro-Wilk test. Moreover, we performed a multivariable analysis considering FORT as a dependent variable and AFC, AFC6, AFC > 6, AMH, BMI, FSH and age as independent variables. The relationship between two continuous variables was assessed by a correlation analysis when they were independent from each other and by regression when there was a dependent relationship between the variables. Spearman’s test was used to determine if the correlation coefficients (r) were significantly different from zero. Considering a correlation coefficient of > 0.3 and a power of 80%, the sample size calculation was 85 patients. A value of *P* < 0.05 was considered statistically significant. The data were analyzed with SPSS 18.0 [SPSS Inc., released 2009, PASW Statistics for Windows, version 18.0, Chicago].

## Results

The study population consisted of 92 women. The characteristics and reproductive outcomes of the patients are shown in Table 1. At the time of inclusion, the age of the women was 36.03 ± 3.87 years (mean ± SD). At baseline, the AFC median was 8.50 follicles (range: 2–25 follicles), AFC6 median was 6.00 follicles (range: 0–25 follicles) and AFC > 6 median was 1.00 follicles (range: 0–11 follicles). Overall, the median FORT was 43.30% (range: 6.67 – 100.00%). A median of 5.00 oocytes (range: 0–30 oocytes) were retrieved. The median AMH level was 1.69 ng/mL (range: 0.09–16.50 ng/mL), and the median FSH level was 6.95 IU/L (range: 1.50–12.50 IU/L). The median BMI was 22.40 kg/m^2^ (range: 19.00–31.20 kg/m^2^).

**Table 1:**
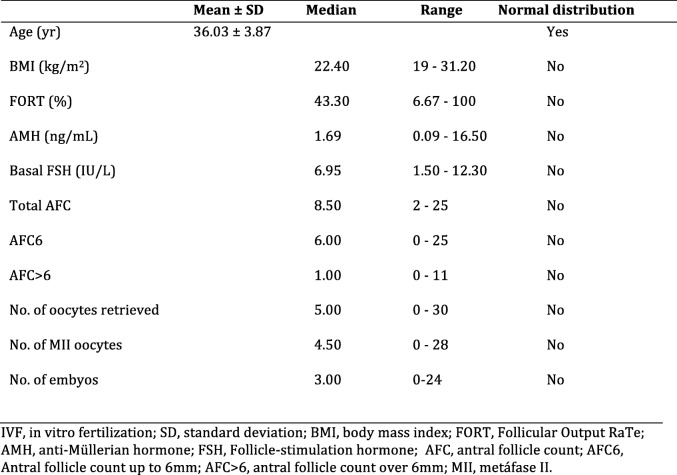
Characteristics and stimulation outcomes from 92 IFV cycles

The indications for IVF-ET were male factor (30.4%), tubal factor (27.2%), multiple causes (19.5%), endometriosis (18.5%) and unexplained infertility (4.3%).

In relation to the gonadotropin dose that was administered at the beginning of the cycle, 75 (81.5%) of the women received the 150 mcg dose of Elonva®, and 17 (18.5%) received the 100 mcg dose. After the seventh day of the Elonva® administration, 35% of the women needed an additional dose of Menopur®. The clinical pregnancy rate was 21 of the 84 who performed embryo transfer (25%).

We analyzed the correlation between the FORT and the diameter of the follicular pool in our main objective, and we also analyzed the correlation between the FORT and other predictors of follicular responsiveness as secondary objectives. The FORT and total AFC and the FORT and AFC6 were inversely correlated at a *P* = 0.05 level of significance (r = − 0.219 and r = − 0.237, respectively). There was no correlation between the FORT and AFC > 6 (r = − 0.055, *P* = 0.602).

As expected, according to our previous study, there was no correlation between FORT and age, FORT and BMI, or FORT and FSH (r = 0.087, P 0.410; r = − 0.152, P 0.174; r = 0.006, P 0.956, respectively). FORT and number of mature oocytes (MII) were correlated (r = 0.270, P 0.011). Table 2 shows the Spearman’s correlation coefficients and *P* values of the analyses.

**Table 2:** Spearman’s Correlation Coefficients among variables and FORT

Variable	R	*P* value
Age	0.087	0.410
BMI	−0.152	0.174
AFC	−0.219	0.036^a^
AFC6	−0.237	0.023^a^
AFC > 6	−0.055	0.602
AMH	−0.102	0.367
FSH	0.006	0.956

FORT, Follicular Output RaTe; BMI, body mass index; AFC, antral follicle count; AFC6, antral follicle count up to 6 mm;AFC > 6, antral follicle count over 6 mm; AMH, anti-Müllerian hormone; FSH, Follicle-stimulation hormone; ^a^ Indicates the correlations with statistical significance

**Fig. 1:**
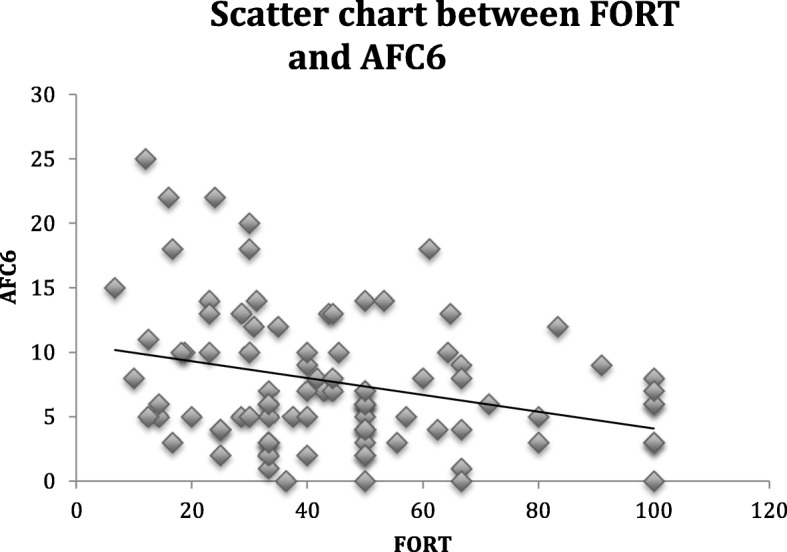
Scatter chart between FORT and AFC6

To verify whether the relationship between the AFC6 and FORT was independent, we performed a multivariable linear regression, adjusting for age, AFC > 6, and AMH, and we found a significative relationship between the two variables (*P* = 0.002), shown in Table 3. Figure 1 shows the scatter chart between FORT and AFC6, demonstrating the inverse relation of those two parameters.

**Table 3:** Multiple linear regression analysis among FORT and the variables

	β-coefficient	95% Confidence Interval		*P*-value
Age	0.268	−1.248	1.784	0.726
AMH	1.758	−0.299	3.816	0.093
AFC6	−2.009	−3.240	−0.777	0.002 ^a^
AFC > 6	−0.309	−2.682	2.065	0.796

FORT, Follicular Output RaTe; AMH, anti-Müllerian hormone; AFC6, Antral follicle count up to 6 mm; AFC > 6, antral follicle count over 6 mm. ^a^ Indicates the correlation with statistical significance

## Discussion

The present investigation aimed to verify whether the responsiveness of antral follicles to gonadotropin is correlated with the size of those follicles. Previous studies have demonstrated that the number of antral follicles up to 6 mm declines with age, differently from the follicles with a diameter greater than 7 mm. Therefore, suggesting that the number of small antral follicles represents the functional ovarian reserve, we divided the total antral follicle count of our cohort in two groups: the follicles with a diameter of 6 mm or less and the follicles with a diameter > 6 mm to compare their responsiveness [14, 19].

The FORT was positively related to the outcomes of IVF, including pregnancy, in normo-cycling women [12, 13], and it can be considered a qualitative marker of follicular responsiveness. Despite several studies having demonstrated a significant predictive value for the antral follicle count in the ovarian response rate and pregnancy rates of patients undergoing IVF treatment [20, 21], there are very few reports in the literature about the predictive value of the antral follicle size in ovarian responsiveness, and none of them have used the FORT as the measure of responsiveness.

We demonstrated that the AFC6 had a negative correlation with the FORT, but there was no correlation between FORT and AFC > 6. From those results, we can suppose that the bigger the number of smaller antral follicles, the lower their sensitivity to exogenous FSH, maybe because of the inhibitory effect of the AMH over those follicles. Also, the absence of correlation between FORT and AFC > 6 indicate that those follicles could be already atretic, which agrees with findings of another study [22].

The findings of our paper are different from the results of Haadsma et al., which show that the follicles under 6 mm are the ones correlated with ovarian responsiveness, but the differential of our study is that we used a better parameter of ovarian response, the FORT, while their study used the clomiphene citrate challenge and inhibin B tests, both of which are no longer used because of their low accuracy in predicting the ovarian response or other reproductive outcomes [23].

The antral follicle count has been used for predicting the ovarian response and for creating an individualized dose adjustment, but a recent multicenter prospective cohort study with two embedded randomized clinical trials failed to demonstrate better live birth rates from an individualized FSH strategy compared to that from a standard FSH strategy based on the AFC results [24]. Perhaps, if only the small antral follicles had been used, the outcome results would have been different.

Results from a study by Lai et al. suggest that the antral follicular size could be a better predictive marker than the basal FSH concentration and BMI during IVF treatment [1]. The same study, in order to assess the impact of antral follicle size on the IVF outcomes, categorized patients into four groups according to the antral follicle size (2–6 mm, 6–7 mm, 7–8 mm and 8–10 mm) and showed that those with an antral follicle size of 6–7 mm had significantly higher AFC, oocyte retrieval and fertilization, and grade I/II embryos compared with the other groups. In addition, there was lower transfer cycle cancellation rate in the group with the antral follicle size of 6–7 mm, despite there being no differences in the implantation rates and clinical pregnancy rates among the groups, probably due to the sample size [1].

Some limitations of this study need to be highlighted, such as the absence of the power to correlate the antral follicle size with the main outcomes of interest in human reproduction, the pregnancy and live birth rates, and the fact that an interobserver difference in ultrasound measures can exist, especially because we used manual bidimensional ultrasound equipment. Additionally, studies with novels ultrasound technologies and among other populations, such as those of polycystic ovaries patients and those with different IVF stimulation protocols, are necessary to endorse the use of the antral follicle size as a predictor of ovarian responsiveness [25].

In light of the lack of evidence about the rule of follicular size over the COH, this is one more indication of its importance, and additional clinical and basic studies are needed to challenge the present results.

## Conclusions

In conclusion, the present findings indicate that ovarian responsiveness, herein represented by the FORT, is inversely correlated with smaller antral follicles, which may express the pool of follicles that are less susceptible to FSH stimulation, in a prospective cohort of normo-cycling women. This is a pioneer result that should be verified in a clinical trial and could provide additional information about the prediction of ovarian responsiveness and a more individualized treatment for patients starting COH.
